# Supplementary material for: ReporterSeq reveals genome-wide dynamic modulators of the heat shock response across diverse stressors
Source: eLife. 2021 Jul 5;10:e57376. doi: 10.7554/eLife.57376 (PMC8257254; doi:10.7554/eLife.57376)
Supplement: Supplementary file 11. [file elife-57376-supp11.zip › library_sequences.docx]

GFP sequence

Actin terminator with random barcodes (NNNNNN)

sgRNA locus (NNNNNNNN)

AmpR cassette

URA3 promoter + URA3 CDS

**Fragment A sequence:**

GGCATGGATGAACTATACAAATAATAAAGATCTT**NNNNNNNNNNNNNNNNNNNNNNNNN**agataaTCTCTGCTTTTGTGcgcgcatgtttatgtatgtacctctctctctatttctatttttaaaccaccctctcaataaaataaaaataataaagtatttttaaggaaaagacgtgtttaagcactgactttatctactttttgtacgttttcattgatataatgtgttttgtCTCTCCCTTTTCTACGAAAATTTCAAAAATTGACCAAAAAAGGGAATATATATACGAAAAACTATTATATTTATATATCATAGTGTTGATAAAAAATGTTTATCCATTGGACCGTGTATCATATGGAATTCTCTTTGAAAAGATAATGTATGATTATGCTTTCACTCATATTTATACAGAAACTTGATGTTTTCTTTCGAGTATATACAAGGTGATTACATGTACGTTTGAAGTACAACTCTAGATTTTGTAGTGCCCTCTTGGGCTAGCGGTAAAGGTGCGCATTTTTTCACACCCTACAATGTTCTGTTCAAAAGATTTTGGTCAAACGCTGTAGAAGTGAAAGTTGGTGCGCATGTTTCGGCGTTCGAAACTTCtccgcagtgaaagataaatgatc**NNNNNNNNNNNNNNNNNNNN**GTTTAAGAGCTAAGCTGGAAACAGCTT

**Fragment B sequence:**

GTTTAAGAGCTAAGCTGGAAACAGCTTAGCAAGTTTAAATAAGGCTAGTCCGTTATCAACTTGAAAAAGTGGCACCGAGTCGGTGGTGCTTTTTTTGTTTTTTATGTCTCTGCAGagttcGGTACCagcttttgttccctttagtgagggttaatttcgagcttggcgtaatcatggtcatagctgtttcctgtgtgaaattgttatccgctcacaattccacacaacatacgagccggaagcataaagtgtaaagcctggggtgcctaatgagtgagctaactcacattaattgcgttgcgctcactgcccgctttccagtcgggaaacctgtcgtgccagctgcattaatgaatcggccaacgcgcggggagaggcggttttcgtattgggcgctcttccgcttcctcgctcactgactcgctgcgctcggtcgttcggctgcggcgagcggtatcagctcactcaaaggcggtaatacggttatccacagaatcaggggataacgcaggaaagaacatgtgagcaaaaggccagcaaaaggccaggaaccgtaaaaaggccgcgttgctggcgtttttccataggctcggcccccctgacgagcatcacaaaaatcgacgctcaagtcagaggtggcgaaacccgacaggactataaagataccaggcgttcccccctggaagctccctcgtgcgctctcctgttccgaccctgccgcttaccggatacctgtccgcctttctcccttcgggaagcgtggcgctttctcaatgctcacgctgtaggtatctcagttcggtgtaggtcgttcgctccaagctgggctgtgtgcacgaaccccccgttcagcccgaccgctgcgccttatccggtaactatcgtcttgagtccaacccggtaagacacgacttatcgccactggcagcagccactggtaacaggattagcagagcgaggtatgtaggcggtgctacagagttcttgaagtggtggcctaactacggctacactagaaggacagtatttggtatctgcgctctgctgaagccagttaccttcggaaaaagagttggtagctcttgatccggcaaacaaaccaccgctggtagcggtggtttttttgtttgcaagcagcagattacgcgcagaaaaaaaggatctcaagaagatcctttgatcttttctacggggtctgacgctcagtggaacgaaaactcacgttaagggattttggtcatgagattatcaaaaaggatcttcacctagatccttttaaattaaaaatgaagttttaaatcaatctaaagtatatatgagtaaacttggtctgacagttaccaatgcttaatcagtgaggcacctatctcagcgatctgtctatttcgttcatccatagttgcctgactgcccgtcgtgtagataactacgatacgggagggcttaccatctggccccagtgctgcaatgataccgcgagacccacgctcaccggctccagatttatcagcaataaaccagccagccggaagggccgagcgcagaagtggtcctgcaactttatcc

**Fragment C sequence:**

GCTTATCATCGATAAGCTTTTCAATTCATCATTTTTTTTTTATTCTTTTTTTTGATTTCGGTTTCCTTGAAATTTTTTTGATTCGGTAATCTCCGAACAGAAGGAAGAACGAAGGAAGGAGCACAGACTTAGATTGGTATATATACGCATATGTAGTGTTGAAGAAACATGAAATTGCCCAGTATTCTTAACCCAACTGCACAGAACAAAAACCTGCAGGAAACGAAGATAAATCATGTCGAAAGCTACATATAAGGAACGTGCTGCTACTCATCCTAGTCCTGTTGCTGCCAAGCTATTTAATATCATGCACGAAAAGCAAACAAACTTGTGTGCTTCATTGGATGTTCGTACCACCAAGGAATTACTGGAGTTAGTTGAAGCATTAGGTCCCAAAATTTGTTTACTAAAAACACATGTGGATATCTTGACTGATTTTTCCATGGAGGGCACAGTTAAGCCGCTAAAGGCATTATCCGCCAAGTACAATTTTTTACTCTTCGAAGACAGAAAATTTGCTGACATTGGTAATACAGTCAAATTGCAGTACTCTGCGGGTGTATACAGAATAGCAGAATGGGCAGACATTACGAATGCACACGGTGTGGTGGGCCCAGGTATTGTTAGCGGTTTGAAGCAGGCGGCAGAAGAAGTAACAAAGGAACCTAGAGGCCTTTTGATGTTAGCAGAATTGTCATGCAAGGGCTCCCTATCTACTGGAGAATATACTAAGGGTACTGTTGACATTGCGAAGAGCGACAAAGATTTTGTTATCGGCTTTATTGCTCAAAGAGACATGGGTGGAAGAGATGAAGGTTACGATTGGTTGATTATGACACCCGGTGTGGGTTTAGATGACAAGGGAGACGCATTGGGTCAACAGTATAGAACCGTGGATGATGTGGTCTCTACAGGATCTGACATTATTATTGTTGGAAGAGGACTATTTGCAAAGGGAAGGGATGCTAAGGTAGAGGGTGAACGTTACAGAAAAGCAGGCTGGGAAGCATATTTGAGAAGATGCGGCCAGCAAAACTAAAAAACTGTATTATAAGTAAATGCATGTATACTAAACTCACAAATTAGAGCTTCAATTTAATTATATCAGTTATTACCCTCGAGCTAGAAGCTTCTAGAAGCTTCTAGAAGCTTCTAGAAGCTTCTAGAGGATCCCCGTCGAGCAGATCCGCCAGGCGTGTATATAGCGTGGATGGCCAGGCAACTTTAGTGCTGACACATACAGGCATATATATATGTGTGCGACGACACATGATCATATGGCATGCATGTGCTCTGTATGTATATAAAACTCTTGTTTTCTTCTTTTCTCTAAATATTCTTTCCTTATACATTAGGTCCTTTGTAGCATAAATTACTATACTTCTATAGACACGCAAACACAAATACACACACTAAATTAATAATGACCGGATCCAAAGGAGAAGAACTTTTCACTGGAGTTGTCCCAATTCTTGTTGAATTAGATGGTGATGTTAATGGGCACAAATTTTCTGTCAGTGGAGAGGGTGAAGGTGATGCAACATACGGAAAACTTACCCTTAAATTTATTTGCACTACTGGAAAACTACCTGTTCCATGGCCAACACTTGTCACTACTTTCACTTATGGTGTTCAATGCTTTGCTAGATACCCAGATCATATGAAACGGCATGACTTTTTCAAGAGTGCCATGCCCGAAGGTTATGTACAGGAAAGAACTATATTTTTCAAAGATGACGGGAACTACAAGACACGTGCTGAAGTCAAGTTTGAAGGTGATACCCTTGTTAATAGAATCGAGTTAAAAGGTATTGATTTTAAAGAAGATGGAAACATTCTTGGACACAAATTGGAATACAACTATAACTCACACAAAGTATACATCACTGCAGACAAACAAAAGAATGGAATCAAAGTTAACTTCAAAACTAGACACAACATTGAAGATGGAAGCGTTCAACTAGCAGACCATTATCAACAAAATACTCCAATTGGCGATGGCCCTGTCCTTTTACCAGACAACCATTACCTGTCCACACAATCTGCCCTTTCGAAAGATCCCAACGAAAAGAGAGACCACATGGTCCTTCTTGAGTTTGTAACAGCTGCTGGGATTACACATGGCATGGATGAACTATACAAATAATAAA……….gggccgagcgcagaagtggtcctgcaactttatccgcctccatccagtctattaattgttgccgggaagctagagtaagtagttcgccagttaatagtttgcgcaacgttgttgccattgctacaggcatcgtggtgtcacgctcgtcgtttggtatggcttcattcagctccggttcccaacgatcaaggcgagttacatgatcccccatgttgtgaaaaaaagcggttagctccttcggtcctccgatcgttgtcagaagtaagttggccgcagtgttatcactcatggttatggcagcactgcataattctcttactgtcatgccatccgtaagatgcttttctgtgactggtgagtactcaaccaagtcattctgagaatagtgtatgcggcgaccgagttgctcttgcccggcgtcaatacgggataataccgcgccacatagcagaactttaaaagtgctcatcattggaaaacgttcttcggggcgaaaactctcaaggatcttaccgctgttgagatccagttcgatgtaacccactcgtgcacccaactgatcttcagcatcttttactttcaccagcgtttctgggtgagcaaaaacaggaaggcaaaatgccgcaaaaaagggaataagggcgacacggaaatgttgaatactcatactcttcctttttcaatattattgaagcatttatcagggttattgtctcatgagcggatacatatttgaatgtatttagaaaaataaacaaataggggttccgcgACATTTCCCCGAAAAGTGCCACCTGACGTTAAGAAACCATTATTATCATGACATTAACCTATAAAAATAGGCGTATCACGAGGCCCTTTCGTCTTCAAGAATTCTGAACCAGTCCTAAAACGAGTAAATAGGACCGGCAATTCTTCAAGCAATAAACAGGAATACCAATTATTAAAAGATAACTTAGTCAGATCGTACAATAAAGCTTTGAAGAAAAATGCGCCTTATTCAATCTTTGCTATAAAAAATGGCCCAAAATCTCACATTGGAAGACATTTGATGACCTCATTTCTTTCAATGAAGGGCCTAACGGAGTTGACTAATGTTGTGGGAAATTGGAGCGATAAGCGTGCTTCTGCCGTGGCCAGGACAACGTATACTCATCAGATAACAGCAATACCTGATCACTACTTCGCACTAGTTTCTCGGTACTATGCATATgatccaatatcaaaggaaatgatagcattgaaggatgagactaatccaattgaggagtggcagcatatagaacagctaaagggtagtgctgaaggaagcatacgataccccgcatggaatgggataatatcacaggaggtactagactacctttcatcctacataaatagacgcatataagtacgcatttaagcataaacacgcactatgccgttcttctcatgtatatatatatacaggcaacacgcagatataggtgcgacgtgaacagtgagctgtatgtgcgcagctcgcgttgcattttcggaagcgctcgttttcggaaacgctttgaagttcctattccgaagttcctattctctagaaagtataggaacttcagagcgcttttgaaaaccaaaagcgctctgaagacgcactttcaaaaaaccaaaaacgcaccggactgtaacgagctactaaaatattgcgaataccgcttccacaaacattgctcaaaagtatctctttgctatatatctctgtgctatatccctatataacctacccatccacctttcgctccttgaacttgcatctaaactcgacctctacattttttatgtttatctctagtattactctttagacaaaaaaattgtagtaagaactattcatagagtgaatcgaaaacaatacgaaaatgtaaacatttcctatacgtagtatatagagacaaaatagaagaaaccgttcataattttctgaccaatgaagaatcatcaacgctatcactttctgttcacaaagtatgcgcaatccacatcggtatagaatataatcggggatgcctttatcttgaaaaaatgcacccgcagcttcgctagtaatcagtaaacgcgggaagtggagtcaggctttttttatggaagagaaaatagacaccaaagtagccttcttctaaccttaacggacctacagtgcaaaaagttatcaagagactgcattatagagcgcacaaaggagaaaaaaagtaatctaagatgctttgttagaaaaatagcgctctcgggatgcatttttgtagaacaaaaaagaagtatagattctttgttggtaaaatagcgctctcgcgttgcatttctgttctgtaaaaatgcagctcagattctttgtttgaaaaattagcgctctcgcgttgcatttttgttttacaaaaatgaagcacagattcttcgttggtaaaatagcgctttcgcgttgcatttctgttctgtaaaaatgcagctcagattctttGTTTGAAAAATTAGCGCTCTCGCGTTGCATTTTTTGTTCTACAATATGAAGCACAGATGCTTCGTAACAAAGATATGCTATTGAAGTGCAAGATGGAAACGCAGAAAATGAACCGGGGATGCGACGTGCAAGATTACCTATGCAATAGATGCAATAGTTTCTCCAGGAACCGAAATACATACATTGTCTTCCGTAAAGCGCTAGACTATATATTATTATACAGGTTCAAATATACTATCTGTTTCAGGGAAAACTCCCAGGTTCGGATGTTCAAAATTCAATGATGGGTAACAAGTACGATCGTAAATCTGTAAAACAGTTTGTCGGATATTAGGCTGTATCTCCTCAAAGCGTATTCGAATATCATTGAGAAGCTGCAGCGTCACATCGGATAATAATGATGGCAGCCATTGTAGAAGTGCCTTTTGCATTTCTAGTCTCTTTCTCGGTCTAGCTAGTTTTACTACATCGCGAAGATAGAATCTTAGATCACACTGCCTTTGCTGAGCTGGATCAATAGAGTAACAAAAGAGTGGTAAGGCCTCGTTAAAGANCAAGGACCTGAGCGGAAGTGTATCGTACAGTAGACGGAGTATACTAGTATAGTCTATAGTCCGTGGAATTCTCATGTTTGACA
