# Supplementary material for: ReporterSeq reveals genome-wide dynamic modulators of the heat shock response across diverse stressors
Source: eLife. 2021 Jul 5;10:e57376. doi: 10.7554/eLife.57376 (PMC8257254; doi:10.7554/eLife.57376)

Fragment A: generation of random barcodes associated with random sgRNAs

Step 1a: PCR of constant region

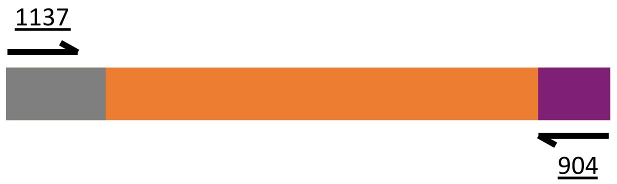

Step 1b: PCR of random sgRNAs

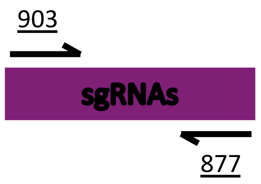

Step 2: PCR of constant region using product of Step 1b as primer

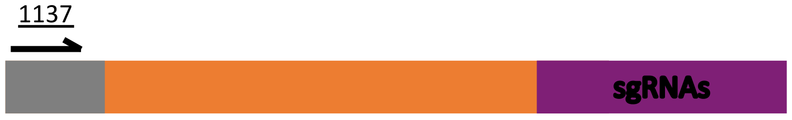

Step 3: PCR to add random barcodes using product of Step 2 as template

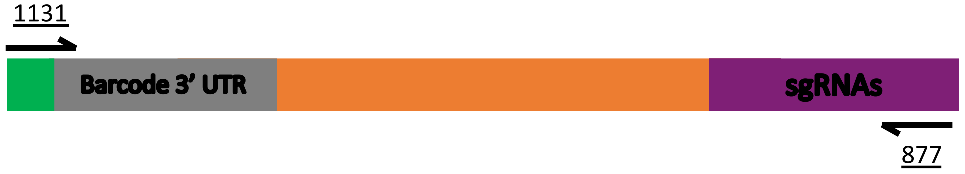

Fragment B: PCR to generate constant region that Connects Fragments A and C

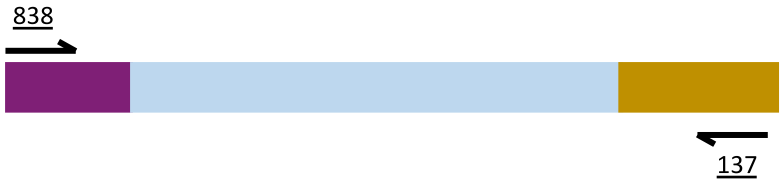

Fragment C: Generation of backbone via digestion with BglI and BglII of parental plasmid containing the reporter

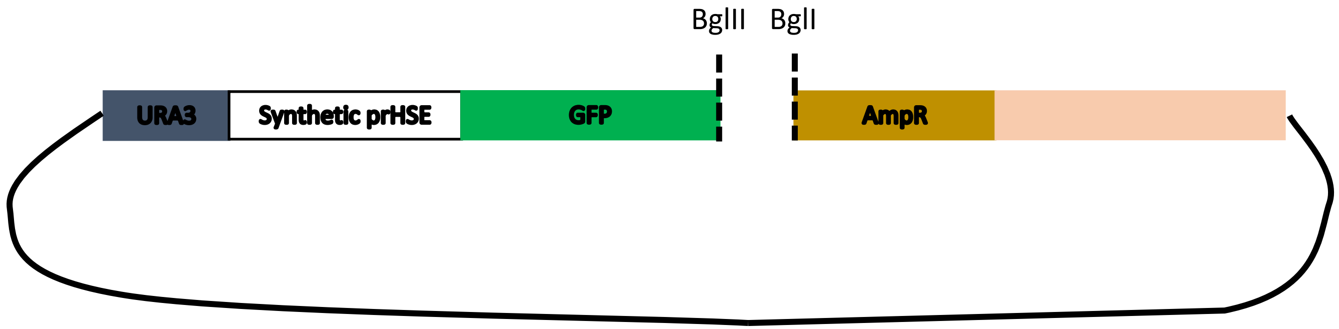

Plasmid assembly of Fragments A, B and C via Gibson Assembly:

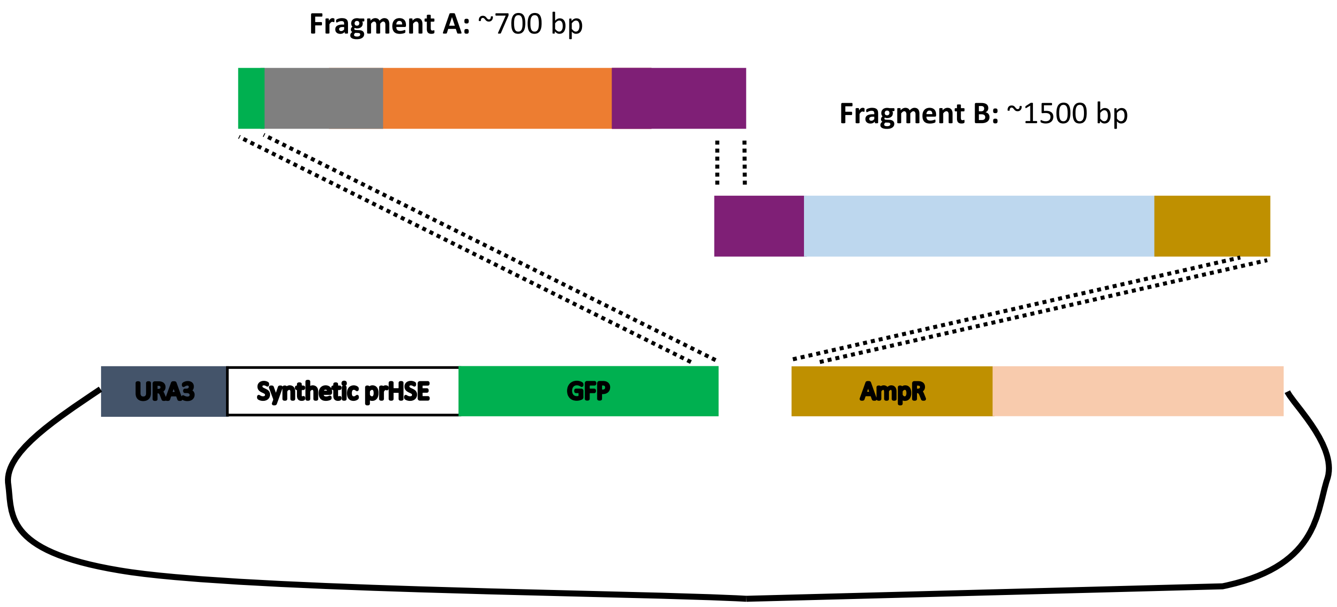

Supplement: Supplementary file 11. [file elife-57376-supp11.zip › library_creation_diagram.pdf]
